# Case Report: Dropped head syndrome as a main clinical sign of suspected myasthenia gravis in two dogs

**DOI:** 10.3389/fvets.2025.1652576

**Published:** 2025-09-26

**Authors:** Henry Mendo Reyes, Greta Galli, Erica Fiorentino, Anna Gardini, Beatrice Bravaccini, Marika Menchetti

**Affiliations:** San Marco Veterinary Clinic and Laboratory, Neurology and Neurosurgery Division, Veggiano, Padua, Italy

**Keywords:** case report, dropped head syndrome, cervical ventroflexion, myasthenia gravis, neuromuscular syndrome, cervical paraspinal muscles, dog

## Abstract

Drop head syndrome (DHS), characterized by pronounced cervical ventroflexion, is a clinical syndrome that can be found associated with neuromuscular disorders, particularly myasthenia gravis. In this case series, we describe two dogs, an 8-year-old Basset Hound and a 3-year-old Zwergpinscher, presenting with DHS as the main clinical sign. In both cases, a presumptive diagnosis of myasthenia gravis was made and were empirically treated with pyridostigmine. Telephone follow-ups at 6 and 4 months after diagnosis, respectively, were consistent with clinical remission. These cases highlight the diagnostic challenges of DHS, emphasizing the need for thorough evaluation to exclude numerous differential diagnoses. In cases where myasthenia gravis is strongly suspected despite negative tests, trial treatment with anti-cholinesterase drugs may be considered, with caution to avoid potential side effects.

## 1 Introduction

Dropped head syndrome (DHS) is a known condition in human medicine, characterized by pronounced flexion of the head and cervical spine, primarily due to weakness of the paraspinal muscles (1). The differential diagnoses are extensive although it is commonly associated with neuromuscular diseases, particularly myasthenia gravis (MG) (2). MG is an autoimmune acquired disease in which autoantibodies are produced against components of the neuromuscular junction of skeletal muscles, such as acetylcholine receptor, muscle-specific kinase, and lipoprotein receptor-related protein 4, among others (3). The binding of these autoantibodies impairs neuromuscular transmission, resulting in skeletal muscle weakness. In some cases, DHS may initially present as an isolated sign that, over time, progresses to a generalized form (4, 5).

In domestic animals, cervical ventroflexion is mainly secondary to neuromuscular diseases, often accompanied by other neurological clinical signs that lead to a neuromuscular localization, such as exercise induced-fatigue, spinal hyporeflexia and flaccid muscle tone, and is most frequently seen in cats due to the absence of nuchal ligament in this species (6, 7). In dogs and cats, MG is classified into three clinical forms: focal, generalized and fulminant, with cervical ventroflexion typically being a feature of the latter two forms (8). To the author's knowledge, severe cervical ventroflexion as main clinical sign, causing a DHS, has not been previously reported as the main manifestation of MG in dogs. Therefore, the aim of this case series is to describe the clinical presentation and outcome of MG-related DHS in two dogs.

## 2 Case description

### 2.1 Case 1

An 8-year-old, 20 kg, spayed female Basset Hound dog was referred to the neurology service of San Marco Veterinary Clinic because a 4-month history of intermittent neck weakness and generalized tremors. Initially, the episodes were triggered by moderate exercise; however, they had progressively worsened, and the dog now exhibited neck ventroflexion after just a few steps.

**Figure 1:**
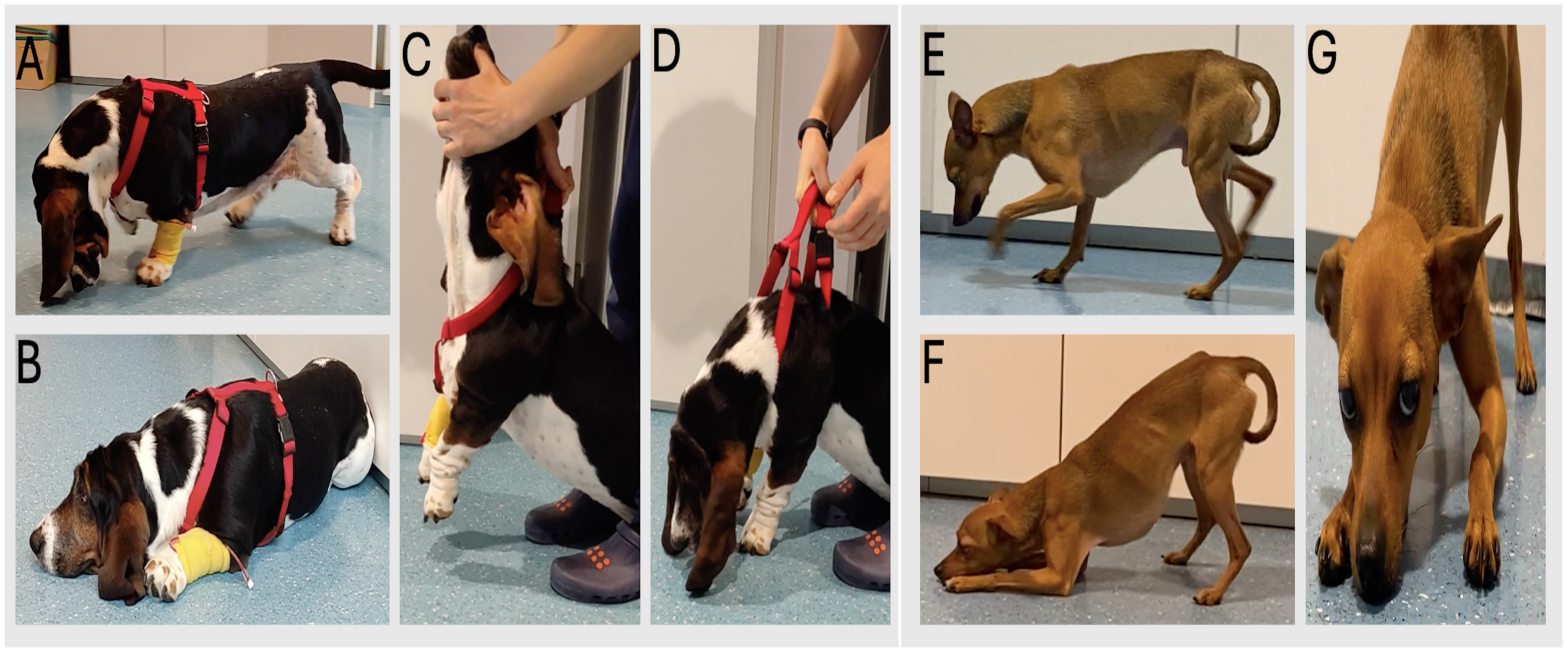
**(A–D)** case 1 with cervical ventroflexion and exercise induced-fatigue. **(C, D)** sequential images illustrating passive cervical ventroflexion. **(E–G)** case 2: note the marked cervical weakness, causing the dog rests its head on the floor.

Physical examination was unremarkable. Neurological examination revealed exercise-induced cervical ventroflexion and fatigue (Figures 1A–D). The neck weakness was so severe that the dog was forced to rest her head on the floor before being able to lift it and walk again (Supplementary Video 1). The remaining neurological examination was normal.

Neuroanatomical localization was neuromuscular system. Main differential diagnoses included inflammatory-non-infectious/immune-mediated diseases, such as myasthenia gravis or polymyositis. Other differential diagnoses were considered less likely based on the neurological findings.

Complete blood work, urine analysis and thoracic radiographs were performed and were unremarkable. *Toxoplasma gondii* serology, assessed by indirect immunofluorescence, was negative. *Neospora caninum* serology revealed a negative IgG titer and an elevated IgM titer of 1:320. A follow-up *Neospora* serology test performed 3 weeks later showed an IgG titer of 1:160 and IgM titer of 1:80. These results were considered irrelevant due to the low antibody titers and the absence of seroconversion, defined as a 4-fold rise in antibody levels.

The dog underwent a whole-body computed tomography (CT) scan (128 × 2 Dual Source CT, Somatom Definition Flash or 192 × 2 Somatom Force, Siemens, Erlangen, Germany), a complete electrodiagnostic study (Nemus2, EBN, Firenze, Italy) consisting of electromyography (EMG), motor nerve conduction study (MNCS), repetitive nerve stimulation (RNS) and F-waves evaluation. Additionally, an anti-acetylcholine receptor (AChR) antibody testing was performed.

CT showed no significant abnormalities.

EMG, MNCS tested at sciatic-tibial and ulnar nerves, RNS and F-waves were normal.

The AChR antibody testing was within normal limits.

Based on the diagnostic test results, a definitive diagnosis could not be established. Due to the high suspicion of myasthenia gravis, a trial treatment with pyridostigmine bromide (Mestinon 60 mg, Viatris, Dublin, Ireland) was initiated at a dose of 0.6 mg/kg PO BID (7), resulting in a partial but clear clinical improvement.

At the 5-day follow-up, the dog continued to exhibit moderate exercise-induced fatigue and cervical weakness. Consequently, the pyridostigmine dose was increased to 0.9 mg/kg BID.

During telephone follow-ups conducted one, seven, and 9 months after the dose adjustment, the owner reported a progressive improvement, with no signs of weakness observed over the previous 2 months. A neurological re-evaluation was recommended but declined by the owner. In agreement with the owner, the pyridostigmine dose was reduced to 0.6 mg/kg BID.

Two months after the last dose adjustment, a follow-up call confirmed that the dog remained clinically normal, prompting a further dose reduction to 0.3 mg/kg BID.

In a subsequent follow-up call 2-months later, the owner had independently discontinued the medication 1 month prior, and the dog remained clinically well.

At the time of the writing, almost 15 months after pyridostigmine discontinuation, the owner continued to report no recurrence of weakness (Supplementary Video 2). A follow-up neurological examination was again offered but declined.

### 2.2 Case 2

A 3-year-old, 4 kg, neutered male Zwergpinscher dog was referred to our institution because a 1-week history of neck weakness.

Physical examination was unremarkable. Neurological examination revealed bilaterally decreased palpebral reflex and persistent neck ventroflexion that markedly worsened with mild exercise, accompanied by generalized muscular weakness (Figures 1E–G). The dog intermittently rested its neck on the floor for several seconds before restarting ambulation (Supplementary Video 1). The remaining neurological examination was normal.

Neuroanatomical localization was neuromuscular system. Differential diagnoses included inflammatory-non-infectious/immune-mediated diseases, such as myasthenia gravis or polyradiculoneuritis. Other differential diagnoses were considered less likely based on the neurological findings.

The diagnostic workup in this case included complete blood analysis, urine analysis, thoracic radiographs, whole-body CT scan and electrodiagnostic studies; the latter two were performed under general anesthesia. Additionally, AChR antibody testing and a neostigmine challenge test were performed.

Complete blood and urine analyses were unremarkable. Thoracic radiographs showed a diffusely dilated esophagus. The CT scan revealed only the presence of megaesophagus. Electrodiagnostic studies, performed using the same protocol as in the first case (EMG, MNCS, RNS and F-waves), yielded normal results. Following administration of neostigmine methylsulfate (0.05 mg/kg intramuscularly; Intrastigmina 0.5 mg/ml, Ist. Luso Farmaco, Milan, Italy), preceded by atropine premedication (one-fourth of the 0.02 mg/kg dose, subcutaneously; Atropina solfato 0.5 mg/ml, Salf, Bergamo, Italy) (9), the dog exhibited a clinical response, transiently maintaining a normal head position for several seconds. AChR antibody titers remained within reference limits.

As in the first case, a definitive diagnosis could not be established; however, a presumptive diagnosis of myasthenia gravis was made. Consequently, a trial treatment with pyridostigmine bromide (Mestinon 60 mg) was initiated at a dose of 1 mg/kg PO BID. Two days after starting therapy, the dog showed a clear improvement with a noticeable reduction in cervical weakness (Supplementary Video 3), Therefore, the patient was discharged with the same pyridostigmine dose. Moreover, appropriate supportive care was recommended to prevent complications secondary to the megaesophagus. This included the administration of a semi-solid diet, increasing feeding frequency to 3–4 times per day, feeding in an upright position, ideally using a Bailey chair, and maintaining that posture for at least 15 minutes after each meal.

At the three- and 8-weeks follow-up, the dog showed progressive neurological improvement, cervical weakness was no longer evident, but a bilaterally reduced palpebral reflex persisted. Additionally, follow-up thoracic radiographs at both time points continued to show the presence of megaesophagus. In agreement with the owner, an increase in the pyridostigmine dose to 1 mg/kg TID was done.

During a telephone follow-up conducted 2 months after the last therapy adjustment, the owner reported that the dog was in good health and exhibiting normal activity.

At the time of the writing, almost 24 months after the last dose adjustment, the owner had independently tapered the pyridostigmine over the previous 6 months. The dog was no longer taking medication for the last 2 months, with no recurrence of clinical signs.

## 3 Discussion

MG is defined as skeletal muscle weakness and fatigability caused by autoantibodies against components of the neuromuscular junction (NMJ).

In dogs and cats, MG is classified into focal, generalized, and fulminating forms. These can be further subclassified based on the presence of a thymoma or other neoplasia, adverse reaction to thiourylene medication in hyperthyroid cats (restricted to the generalized form), and seronegative MG in dogs (also limited to the generalized form) (8). Whole-body CT is recommended as part of the diagnostic workup of MG to investigate potential underlying neoplastic triggers.

Focal MG is defined as weakness affecting one or more focal skeletal muscle groups without involvement of the appendicular skeletal muscles. These focal groups may involve the facial, esophageal, pharyngeal and laryngeal muscles. In contrast, generalized MG affects the appendicular skeletal muscles, while the fulminant form represents a severe, acute, and rapidly progressive variant of generalized MG (8). In this case series, we describe two dogs presenting with the generalized form of MG, both exhibiting marked weakness of the neck extensor muscles, resulting in pronounced cervical ventroflexion. In the first case, DHS was the predominant manifestation, triggered by mild exercise, with only mild to moderate appendicular muscle involvement. In the second case, DHS was persistent and exacerbated by exercise, occurring alongside facial and esophageal muscle involvement and exercise-induced fatigue.

DHS is typically painless and passively correctable, and must be differentiated from low head carriage, which is an antalgic posture, usually secondary to cervical pain. In humans, DHS has a broad differential diagnosis, including myasthenia gravis, polymyositis, isolated neck extensor myopathy, hypokalemic myopathy, amyotrophic lateral sclerosis, and chronic inflammatory demyelinating polyneuropathy, among others (2, 10, 11). Particularly in MG, DHS may initially be the sole clinical sign and can later progress to a generalized form; therefore, a thorough diagnostic work-up is essential to exclude other differential diagnoses (4, 5). In contrast, the differential diagnosis of DHS in dogs is more limited, primarily involving neuromuscular disorders, while central nervous system diseases, particularly cervical myelopathies, and certain toxicities (e.g., organophosphate poisoning) are considered uncommon causes in this species (12).

Common diagnostic findings supportive of MG include neurological signs such as exercise-induced fatigue, electrophysiological evidence of a decremental response to RNS, a positive response to a short-acting acetylcholinesterase inhibitor (edrophonium challenge test), and elevated AChR antibody titers. Although edrophonium is no longer commercially available, neostigmine has been investigated and proposed as a suitable alternative for the challenge testing (9). In our case series, however, aside from the neurological examination, the remaining diagnostic tests did not support a diagnosis of MG. In human medicine, single-fiber EMG has demonstrated higher sensitivity than RNS for both generalized and ocular MG (13, 14); nonetheless, due to technical complexity and limited expertise in veterinary medicine, it is not routinely performed.

In human medicine, several autoantibodies associated with MG have been identified, including those targeting the AChR, muscle-specific kinase (MuSK), lipoprotein receptor-related protein 4 (LRP4), agrin, titin and ryanodine receptors (3). In veterinary medicine, autoantibodies most frequently target the AChR, and the measurement of AChR antibody titers is considered the gold standard for diagnosis. Although autoantibodies against other components of the NMJ have also been reported in dogs, there are currently no available tests for their detection, nor established reference ranges (8). In the two cases presented here, the AChR antibody titers were within reference limits, and neither patient had received prior corticosteroid or immunosuppressive therapy that could have interfered with the results. Based on the clinical presentation and positive response to pyridostigmine, we concluded that both cases were compatible with seronegative MG; however, they did not fully meet the established criteria for this categorization by Mignan et al., as a second negative AChR titer measurement was not obtained (8).

In the first case, polymyositis was considered a major differential diagnosis because it can present with tetraparesis mimicking exercise-induced fatigue, and may affect multiple skeletal muscles, including cervical extensor muscles, potentially leading to DHS (7). It may also occasionally show a positive response to neostigmine challenge (9). However, the absence of elevated muscle enzyme levels, the normal EMG findings, and the clear clinical response to pyridostigmine alone, without the use of immunosuppressive therapy, made polymyositis an unlikely diagnosis.

In the second case, polyradiculoneuritis was included among the main differential diagnoses because it typically causes tetraparesis or tetraplegia, reduced or absent spinal reflexes, and muscle hypotonia, which may result in DHS when the cervical muscles are affected. Some patients may also exhibit facial weakness and, less commonly, megaesophagus (7). Nevertheless, in our case, spinal reflexes and electrodiagnostic studies were normal, and the rapid and sustained clinical improvement with pyridostigmine made polyradiculoneuritis unlikely.

The primary treatment for canine MG involves acetylcholinesterase inhibitors (AChEi) such as pyridostigmine, which enhance neuromuscular transmission by increasing acetylcholine availability at the NMJ. These agents provide symptomatic relief and allow time for potential spontaneous immunological remission. However, they must be used cautiously to avoid cholinergic crisis (e.g., hypersalivation, diarrhea, muscle fasciculations). Supportive care is also crucial, especially in cases presenting with megaesophagus, to minimize the risk of aspiration pneumonia. In cases with inadequate response to AChEi therapy and/or significant side effects upon dose escalation, immunosuppressive therapy should be considered. In the two cases presented, the final telephone follow-up suggested that both dogs achieved likely immunological remission.

The prognosis for MG in dogs is variable. Reported mean times to clinical and immunological remission are approximately 4.1 and 6.4 months, respectively (15). The presence of megaesophagus and/or thymoma typically are associated with worse outcomes. Interestingly, despite the presence of megaesophagus in case 2, the condition appeared clinically silent, as the owner did not report vomiting or regurgitation. This absence of overt clinical signs may have contributed to the favorable outcome.

This study has several limitations. First, the small number of cases limits the generalization of these findings to the general canine population; however, case reports remain valuable for advancing medical knowledge, particularly in cases with diagnostic challenges such as those presented here. Second, a definitive diagnosis of MG was not achieved despite an extensive diagnostic work-up. Muscle and/or nerve biopsies could have confirmed or excluded other neuromuscular disorders but given the absence of abnormalities in the tests performed and the marked positive response to AChEi, we considered seronegative MG highly likely and deemed biopsy unnecessary. Finally, long-term follow-ups were mainly conducted by telephone, which may have affected the accuracy of outcome assessment, although the inclusion of video recording, at least in the first case, partially mitigated this limitation.

In conclusion, DHS in dogs primarily suggests an underlying neuromuscular disorder, and an exhaustive diagnostic approach is essential to exclude many differential diagnoses. In cases where MG is strongly suspected despite negative tests, a therapeutic trial with anticholinesterase agents may be considered with appropriate caution to mitigate the risk of side effects.

## Data Availability

The original contributions presented in the study are included in the article, further inquiries can be directed to the corresponding author.
